# Clowning in care: long-term effectiveness of clown visits on residents’ affect, resilience, sleep quality and functionality

**DOI:** 10.1186/s12877-026-07874-0

**Published:** 2026-06-29

**Authors:** Laura Korock, Tabea Scheel

**Affiliations:** Department of Work and Organisational Psychology, Europa-Universität Flensburg, Munketoft 3b, Flensburg, 24937 Germany

**Keywords:** Therapeutic clowning, Long-term care, Humour-based intervention, Affect, Resilience, Sleep quality, Functional outcomes

## Abstract

**Background:**

Clown visits are increasingly used as psychosocial interventions in long-term care settings. However, existing evidence is predominantly qualitative and/or short-term, with a lack of controlled longitudinal studies examining effects across affective, resilience-related, sleep-related, and functional outcomes. Moreover, the role of individual-based visit frequency remains largely unexplored.

**Methods:**

This longitudinal controlled field study examined the effects of clown visits over two years in four long-term care facilities in Eastern Germany. The intervention group comprised *n*_*IG_T0*_ = 135 residents at baseline, *n*_*IG_T1*_= 163 after one year, and *n*_*IG_T2*_= 121 after two years; the control group included *n*_*CG_T0*_= 27, *n*_*CG_T1*_= 50, and *n*_*CG_T2*_= 43 participants. An additional waiting control group included *n*_*WGC_T−1*_= 43 residents assessed prior to intervention onset. Validated self- and proxy-report measures assessed affect, resilience, sleep quality, and functionality. Multilevel models were used to analyse group differences in change over time and to test individual-based visit frequency as a moderator; exploratory multilevel analyses distinguished within-person and between-person associations.

**Results:**

After one year, residents receiving clown visits showed more favourable developments in sleep-related outcomes and depressive mood than the control group. After two years, the declines in positive mood and resilience were significantly less pronounced in the intervention group. No consistent effects emerged for other functional domains, and visit frequency did not moderate longitudinal change. Exploratory analyses indicated that, within individuals, higher visit frequency was associated with greater self-care difficulties, disorientation, withdrawal, lower resilience, and higher daytime sleepiness; between individuals, it was linked to higher disorientation, positive affect, and lower negative affect.

**Conclusion:**

Clown visits showed time-specific benefits for residents’ mood, sleep quality, and resilience - without adverse effects. Their contribution appears to lie particularly in supporting residents’ emotional well-being and sustaining resilience over time, rather than broadly altering functional trajectories. The pattern of visit frequency underscores an adaptive delivery aligned with residents’ psychosocial and functional needs, highlighting clown visits as a flexible, context-sensitive intervention embedded in everyday care. As such, clown visits may contribute to preventive care and supportive emotional climate in long-term care, with potential benefits for nursing practice.

**Trial registration:**

Not applicable.

**Supplementary Information:**

The online version contains supplementary material available at 10.1186/s12877-026-07874-0.

## Introduction

The WHO highlights that population ageing increases the global demand for long-term care [1]. This trend poses significant challenges to healthcare systems worldwide, including shortages of qualified professionals and escalating healthcare costs, both of which threaten the quality of care provision [2]. In response, the WHO launched the ‘UN Decade of Healthy Ageing 2021–2030’, emphasising the need to improve the quality of long-term care and to promote interventions that enhance emotional well-being and social participation among older adults [1].

The German long-term care sector reflects these global developments. Despite a comprehensive legal framework aimed at ensuring high standards, persistent staff shortages, overburdened personnel, and limited time resources continue to restrict caregivers’ ability to address residents’ emotional and social needs [3, 4]. Accordingly, a meta-analysis of international studies reports high prevalence rates of loneliness in residential care, with 61% of residents experiencing moderate and 35% severe loneliness [5]. Beyond loneliness, residents often experience reduced autonomy due to rigid daily routines [6], age-related health decline, and frequent confrontations with loss and death [7, 8]. The COVID-19 pandemic further exacerbated these challenges, as prolonged restrictions in care homes limited engagement and participation. The results were lasting declines in psychological and social well-being [9, 10], including increased depression and anxiety [11], and accelerated cognitive and physical health deterioration [9].

To address these cognitive, emotional, and social challenges residents face, various psychosocial interventions, such as reminiscence or music therapy, have been introduced in long-term care settings. While these approaches show initial effectiveness in enhancing cognitive functioning and general quality of life, particularly among residents with dementia (e.g [12]), evidence regarding their impact on depressive mood and broader psychosocial well-being remains limited and inconsistent [12, 13]. In addition, the overall evidence base is weakened by substantial methodological heterogeneity, limiting the robustness and generalisability of existing findings [13, 14]. This highlights a broader research gap regarding interventions targeting not only depressive mood but also residents’ functionality and resilience.

In this context, clown visits offer a promising alternative. These interventions combine humour, empathy, and individual-tailored, playful interactions to foster moments of joy and self-directed social engagement, providing relief from isolation [15]. Although research has largely focused on paediatric healthcare settings [16], emerging qualitative and quantitative findings suggest that clown visits may also benefit older adults by enhancing mood and reducing agitation [17, 18]. However, the existing evidence base in elderly care remains narrow, predominantly qualitative, and short-term (maximum six months; e.g. [6, 19, 20]). To date, long-term effects on broader well-being outcomes, such as two years, have not been examined.

This study addresses this gap by evaluating the long-term effects of clown visits on affect, resilience, sleep quality and functionality in elderly care residents. In doing so, this quantitative study contributes to the growing body of research on humour interventions as a prevention tool in care settings, answering the following question:

*How do clown visits and their individual-based frequency per year influence affect*,* resilience*,* sleep quality and functionality (including self-care and depression) among elderly residents in long-term care facilities?*

Our evaluation follows a multigroup longitudinal design, with three time points (T_0_ = pre-intervention, T_1_ = one year with intervention, T_2_ = two years with intervention), comparing three intervention groups (IG) with a control group (CG) and a waiting control group (T_− 1_ = three months before intervention to T_0_). By adopting a two-year longitudinal approach, the study contributes to the limited empirical literature on clown visits in elderly care and advances understanding of humour-based interventions in the context of ageing.

### Clown visits and its mechanisms

Clowns in health and care settings are specially trained to engage residents through humour, improvisation, and empathy, fostering playful moments that unite physical, intellectual, emotional, and spiritual aspects [21]. In Germany, visits are not part of the regular healthcare system and are usually organised by non-profit organisations. Clowns typically work in pairs, with visits in care facilities scheduled weekly or fortnightly (e.g [22]).

The effects of clown visits may be understood through the Broaden-and-Build Theory [23, 24], which posits that positive emotions broaden thought-action repertoires and build resources such as resilience. Clown visits may evoke positive emotions like joy, cheerfulness, and surprise in residents (e.g [15, 25]), which could foster short-term gains in openness, social interaction, cognitive flexibility, and emotional regulation [23, 24]. Over time, these immediate effects may accumulate into enduring social, intellectual, physical, and psychological resources, thereby strengthening coping behaviour and potentially initiating upward spirals of positive emotions [26, 27]. The regularity of visits may reinforce this process by continuously sparking positive affect and sustaining long-term improvements.

In addition to emotional mechanisms, residents’ physiological responses to laughter represent another pathway of influence. Laughter activates parasympathetic processes that reduce muscle tension, promote cardiovascular health [28], enhance pain tolerance through endorphin release [29], lower cortisol levels and thus reduce stress and anxiety [30, 31], and boosts immune function by increasing lymphocyte activity [32]. Clown visits can elicit laughter in response to humour and play [25], thereby possibly contributing to both mental and physical well-being.

Beyond physiological effects, clown visits also offer a distinct psychosocial space that addresses key resident’s needs, such as the ability to impact their environment, meaningful, barrier-free social interaction or opportunities for reminiscence [6, 33]. As shown in a qualitative study on hospitalised children [34], a special bond may also develop between clowns and residents in care facilities, offering residents opportunities to step outside rigid routines and demands of daily life. The anonymity of the clowns enhances a sense of security and freedom, enabling residents to engage in the interactions without the pressure of personal expectations [34]. Such shared moments may not only foster light-heartedness and joy but also create a space for processing difficult topics. Humorous communication and playful interactions also encourage cognitive reframing [35], helping residents approach themes such as loss or loneliness with emotional distance and new perspectives [17].

Finally, clown visits align with the Stress-Buffer Theory, which conceptualises social support as a protective factor against stress [36]. As an additional source of support beyond care staff, clowns may foster social connections and provide emotional relief. Stronger social ties in long-term care are linked to better mental health outcomes, including reduced cognitive decline and more positive affect [37]. By enriching the social environment and reducing emotional strain, clown visits may thereby enhance residents’ humour, mood, and overall emotional and social well-being.

### Empirical evidence for the effects of clown visits

The idea that clown visits can positively affect psychological and physical well-being has mainly been studied with children in hospitals. Research shows that clown visits can increase happiness, cheerfulness, mood, and life satisfaction in children and their parents [16, 38]. Meta-analyses also indicate reductions in stress and anxiety levels in both children and parents [39, 40], as well as reduced pain and crying time in children, leading to shorter hospital stays [41, 42].

In nursing homes, evidence remains limited and largely qualitative (e.g [6, 19, 20]). The few quantitative studies suggest that clown visits can likewise increase residents’ happiness [18] and reduce dementia-related agitation [18, 43]. Recent qualitative findings from Germany show that clowns can, in rare cases, engage residents who are otherwise difficult to reach and may even temporarily reactivate abilities such as speech or motor skills [17]. More commonly, residents described the visits as the highlight of the day, offering a break from daily routines and fostering positive social interactions. The clowns’ relational work often improved the atmosphere in the facility, eliciting joy, emotional relief, and visible physical relaxation. Overall, these qualitative findings highlight the multidimensional benefits of clown visits for residents’ physical and emotional well-being.

Despite these promising findings, there is limited evidence on the long-term impact of clown visits to nursing homes, as most studies only cover three to six months (e.g [18, 43]). Our study aims to examine these results quantitatively by investigating the effects of clown visits on residents’ affect, resilience, sleep quality and functional ability, with individual-based visit frequency per year serving as a moderating factor.

#### Affect

Referring to the general pattern of positive and negative emotional experiences over a certain period of time, affect reflects the frequency and intensity of discrete emotions [44]. High positive affect and low negative affect are central indicators of subjective well-being. In long-term care, positive affect tends to decline, particularly in residents with dementia, while negative affect remains relatively stable [45, 46].

Nevertheless, affect is considered malleable and responsive to interventions. For example, participation in activity programmes has been shown to enhance positive affect in nursing home residents [47], and an eight-week humour intervention increased positive and reduced negative affect in Chinese nursing home residents [48]. Individualised and preference-based activities appear especially effective in promoting positive affect [49], which aligns with the personalised nature of clown visits. Although most evidence on clown interventions stems from paediatric care, these studies consistently report benefits for emotional states (e.g [16, 38]). Based on this evidence, we expect that positive affect will decline less, and negative affect will decrease more, in residents receiving clown visits.

- H_1_: The (a) positive affect will decline less in the intervention group (IG) than in the control group (CG), and (b) negative affect will decrease in the IG compared to the CG, over (i) one year (T_1_) and over (ii) two years (T_2_).

#### Resilience

As a personal resource, resilience reflects the individual’s ability to cope with stress and regulate emotions in facing adversity [50]. For residents, resilience plays a crucial role in adapting to physical, mental and social changes associated with ageing, as well as stressors and conflicts in long-term care facilities, thereby helping to maintain their quality of life [7, 51]. Higher resilience is associated with lower levels of depression and anxiety [52], greater happiness, and better psychological well-being [53]. However, resilience also tends to decline with age due to worsening health, cognitive impairments, and social losses [7, 54, 55].

Resilience develops through genetic, biological, psychological, and environmental factors [56] and can be modified and improved through treatment [7, 57, 58]. In line with the Broaden-and-Build theory [23, 24], positive emotions and perceived social support have been shown to foster resilience [59–61]. Since positive humour has also been identified as a predictor of resilience [62, 63], clown visits may help residents experience positive emotions and supportive social interaction, thereby contributing to maintaining resilience over time.

- H_2_: The resilience of elderly residents declines less in the IG compared to the CG over (i) one year and over (ii) two years.

#### Sleep quality

Sleep is a fundamental component of physical and mental health, particularly in older adults, where disturbances are common and can substantially affect well-being [64, 65]. With increasing age, sleep becomes less efficient, characterised by shorter duration, greater fragmentation, and difficulties initiating sleep, which contribute to increased daytime sleepiness and are often further exacerbated by comorbidities such as dementia, depression, cardiovascular disease and polypharmacy [66, 67]. Moreover, sleep quality can be shaped by emotions and interact with physical and social factors [68].

Non-pharmacological interventions, including physical activities, sensory stimulation and psychological approaches, can improve subjective sleep quality, for instance by reducing daytime napping [69]. Initial studies on humour interventions, including a four-week laughter therapy and an eight-week humour training programme, have also reported improved sleep quality [48, 70]. However, psychological interventions addressing sleep quality remain underexplored in older adults [65], and to our knowledge no study has examined whether clown visits, as a combined physical and social activity, may positively influence residents’ sleep patterns over time.

- H_3_: The sleep parameters (a) sleep disturbance, and (b) daytime sleepiness increase less in the IG compared to the CG, while (c) overall sleep quality decreases less in the IG than in the CG over (i) one year and over (ii) two years.

#### Functionality

Functionality refers to an individual’s ability to perform daily activities independently, making it a key indicator of quality of life, health, and autonomy in old age [71]. In geriatric assessments, functionality encompasses five behavioural domains that reflect physical, cognitive, and psychosocial functioning: self-care difficulties (required assistance for dressing, bathing, and grooming), disoriented behaviour (difficulties with memory or spatial and temporal orientation), depressed or anxious mood (emotional well-being and depressive symptoms), irritable behaviour (emotional reactivity and frustration), and withdrawal behaviour (social engagement and ability to maintain social connections; [71]).

Functional abilities typically decline with age [7, 72, 73] and can be affected by health limitations, cognitive decline or social isolation [74]. Functional losses, such as language impairments or motoric difficulties, can further hinder individuals’ ability to engage in social interactions, contributing to social isolation and feelings of loneliness [75]. Evidence suggests that positive emotions are associated with better physical health and improved social interactions [76], which in turn may help maintain functionality. Humour has also been shown to enhance functionality in nursing home residents, with intervention studies reporting that humour training and therapy can reduce depression and anxiety while improving subjective well-being and cognitive function [22, 48]. Based on these findings, clown visits could foster emotional responses and social engagement, potentially slowing the declines in residents’ functionality.

- H_4_: The functionality of elderly residents [a) self-care difficulties, b) disorientation, c) depression, d) irritability, e) withdrawal] declines less in the IG compared to the CG over (i) one year and over (ii) two years

#### Frequency of clown visits

The frequency of clown visits and its impact on effectiveness have been scarcely explored to date. A paediatric study suggests that the effects of clown visits were not significant after a single visit but became evident with regularity [77]. Similarly, qualitative findings from care facilities further indicate that higher visit frequency facilitates relationship-building, particularly among residents with dementia, and that weekly visits may be especially supportive for establishing meaningful connections [17]. According to the Broaden-and-Build Theory [23, 24], frequent visits provide more opportunities to evoke positive emotions, thus strengthening the long-term development of psychological resources. Based on this, we expect that individual-based frequent clown visits will strengthen the intervention’s effectiveness.

- H_5_: The effects of clown visits on residents’ (a) affect, (b) resilience, (c) sleep quality and (d) functionality are moderated by the frequency of visits per year, with a higher frequency of visits associated with stronger positive effects.

## Methods

### Study design and sample

**Fig. 1:**
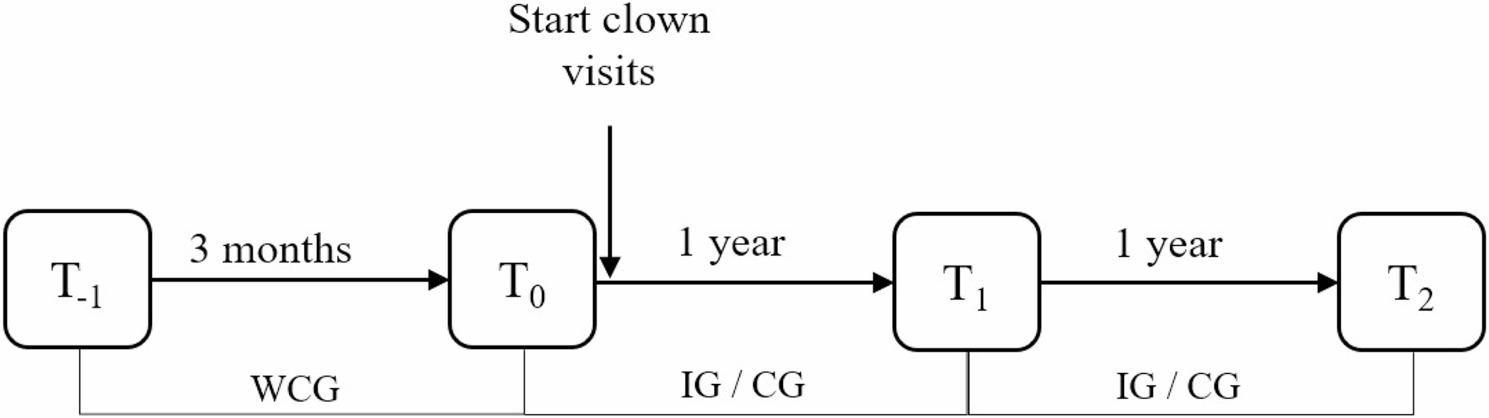
Data collection process. T-_1_ = pre-intervention assessment (three months prior), T_0_ = baseline assessment, T_1_ = 1-year follow-up, T_2_ = 2-year follow-up. IG = intervention group, CG = control group, WCG = waiting control group

This quantitative study followed a multigroup longitudinal design with one IG, one CG, and one waiting control group (WCG). Data were collected at four time points: T_− 1_ (only WCG, three months pre-intervention), T_0_ (baseline), T_1_ (one year with intervention), and T_2_ (two years with intervention; see Fig. 1).

The study took place in four nursing homes in Eastern Germany between August 1, 2022, and March 31, 2025, with three homes receiving clown visits (= IG) and one home serving as the control group (= CG). In addition, one of the intervention homes was assessed three months before the start of the intervention (T_− 1_), serving as a WCG before joining the IG at T_0_. The study was conducted in collaboration with AOK-Plus - Gesundheitskasse für Sachsen und Thüringen, a health insurance provider that funded the project, and the clown organisation Rote Nasen Deutschland e. V., which carried out the clown visits.

All residents were recruited without exclusion criteria. Residents were invited to participate by the care home management, and their relatives were informed and asked to provide consent. Both residents and relatives received an information sheet describing the project’s purpose and procedures, the voluntary nature of participation, data anonymity and protection (including individual resident codes for linking data across time points), and the option to withdraw at any time without consequences. Data were collected using paper-and-pencil questionnaires. The IG included *n*_*T0*_= 135, *n*_*T1*_= 163, *n*_*T2*_= 121, the CG *n*_*T0*_= 27, *n*_*T1*_= 50, *n*_*T2*_= 43, and the WCG *n*_*T−1*_= 43 participants. At T_1_, which comprised the largest sample, the IG had a mean age of *M* = 84.44 (*SD* = 8.07) and the CG of *M* = 87.04 (*SD* = 7.69), with a total range of 65 to 106 years. Women constituted 76.7% of the IG (men = 23.3%; others = 0%) and 78.0% of the CG (men = 22.0%; others = 0%). The average care level (i.e. official German “Pflegegrad”; 1 = *minor* to 5 = *severe impairments*) was *M* = 3.59 (*SD* = 0.88) in the IG and *M* = 3.34 (*SD* = 0.75) in the CG. Length of stay averaged *M* = 2.93 years (*SD* = 2.67) in the IG and *M* = 3.77 years (*SD* = 2.23) in the CG, ranging from two weeks to 12.5 years. See Table 4 in the Appendix for a full demographic summary.

This study was approved by the ethics committee of the German Psychological Society (DGPs) in accordance with the ethical principles outlined in the Declaration of Helsinki. It was preregistered on the Open Science Framework (OSF) under osf.io/pje3v (associated with the overall project: osf.io/yef7v), ensuring transparency and reproducibility.

### Clown visits

In the three intervention facilities (IG_1_, IG_2_, IG_3_), clown visits began in IG_1_ and IG_2_ in summer 2022 and in IG_3_ six months later. IG_1_ and IG_3_ received weekly visits from the outset, whereas IG_2_ initially received biweekly visits, which switched to weekly after six months. A total of 28 professionally trained clowns participated in the project, all of whom had completed several years of artistic clown training, additional qualifications for therapeutic and social settings within Rote Nasen Deutschland e. V., and regular medical and psychological training.

Visits were typically conducted in pairs. Pairings changed frequently at the beginning and later stabilised to ensure continuity. A visit lasted around two hours and included up to 20 individual encounters with residents of five to ten minutes each. Although originally designed as one-to-one encounters, interactions often evolved into small-group sessions in hallways, creating a mix of individual and group engagements. Before each visit, clowns received a handover from care staff with health-related updates and had access to a general folder containing residents’ photographs and biographical notes. This preparation enabled them to tailor their interactions through humour, play, music, dance, shared joy and reminiscence work, and to foster personalised relationships characterised by lightness and humour.

Residents’ participation was always voluntary, and they could decline at any time. The clowns actively sought to establish contact and adapted their approach to each resident’s functional abilities, drawing on different sensory modalities such as touch, music, rhythm or movement. They were trained to offer repeated opportunities for engagement while remaining non-intrusive and respecting personal boundaries. Each visit to the facility was followed by a debriefing between the performing clowns and, when relevant, with medical staff. In addition, all project partners met at least once a year at each facility to reflect, coordinate their work and review progress, making adjustments as needed. Beyond the clown visits for residents, the overall-project also offered humour training for care staff to support the long-term integration of humour into organisational culture.

### Operationalisation of variables

#### Affect

The scale of Positive and Negative Experience (SPANE) [44, 78] assesses the frequency of residents’ positive and negative affective experiences over the past four weeks with 12 items (e.g. happy, positive, unpleasant), rated on a 5-point Likert scale (1 = *very rarely or never*, 5 = *very often or always*). Whenever possible, residents answered the items themselves; otherwise, staff responded on their behalf. Cronbach’s α showed acceptable to good reliability in both subscales (IG: α = 0.79 to 0.91; CG: α = 0.63 to 0.90; WCG: α = 0.85 to 0.93).

#### Resilience

Ten items (e.g. “I am able to adapt when changes occur.”) of the Connor-Davidson Resilience Scale (CD-RISC-10) [57] (German version [79]) were assessed on a 7-point Likert scale from *not at all* (1) to *fully* (7) and obtained good to excellent Cronbach’s α (IGs: α = 0.95 to 0.96; CG: α = 0.88 to 0.89; WCG: α = 0.97). Residents completed the self-report items whenever possible; otherwise, staff answered on their behalf.

#### Sleep quality

Five items from the Pittsburgh Sleep Quality Index (PSQI) [80] (German version [81]) were used to assess facets of sleep. Factor analysis showed that the sleep latency items did not form a distinct subscale but instead loaded onto the sleep disturbances factor. Accordingly, three subscales were formed in this study: sleep disturbances (three items; e.g. “During the past month, how often have you had trouble sleeping because you cannot get to sleep within 30 minutes?”; IG: α = 0.78 to 0.87; CG: α = 0.74 to 0.77; WCG: α = 0.63), *daytime sleepiness* (single-item: “During the past month, how often have you had trouble staying awake while eating meals or engaging in social activity?”) and *sleep quality overall* (single-item: “During the past month, how would you rate your sleep quality overall?”). The first four items were rated on a 4-point Likert scale (1 = *not during the past month* to 4 = *three or more times a week*), whereas subjective sleep quality was rated from *very good* (1) to *very bad* (4) and subsequently recoded. Whenever possible, residents answered the items themselves; otherwise, staff responded on their behalf.

#### Functionality

To assess the residents’ functionality, the Multidimensional Observation for Elderly Subjects scale (MOSES) [71] was translated into German using forward-backward translation [82]. Nursing staff proxy-rated residents’ cognitive and psychosocial functioning over the past week. The response format followed a four-point Likert scale ranging from (1) *behaviour is not present or is normal* to (4) *behaviour is very severe or occurs almost constantly*, supported by item-specific examples. For 15 items, a fifth option (5) *does not apply* was available. To minimise missing data, particularly among residents with severe impairments, we followed an established coding procedure [83] and coded this response either as the highest impairment (4) or as not present (1), depending on the behavioural meaning of the item (e.g. speaking does not occur when a resident can no longer speak; see Table 5 in the Appendix).

The 31 items formed five different subscales: self-care difficulties, disorientation, depression, irritability, and withdrawal (e.g. “irritability - How often during the past week was the resident irritable and grouchy?: 1 = *not at all*; 2 = *seldom (on one or three days for only short periods of time);* 3 = *at times (either on more than three days or only short periods of time*,* or on one to three days for most of the day)*; 4 = *often (on more than three days for most of the day)”).* Cronbach’s α showed good reliabilities in all subscales (IG: α = 0.75 to 0.94; CG: α = 0.73 to 0.94; WCG: α = 0.86 to 0.92).

#### Frequency of clown visits

The single-item visit frequency was based on documentation completed by the clowns, in which resident’s code was recorded for each visit, allowing aggregation of visits per resident and year. On average, residents in the IG received *M* = 9.37 (*SD* = 8.64) visits in the first and *M* = 15.92 (*SD* = 11.29) visits in the second year.

#### Control variables

Gender (1 = *woman*, 2 = *man*, 3 = *other*) was included as a covariate (dummy-coded: 0 = *man*, 1 = *woman*), based on findings of gender-related differences in the effects of clown visits [77]. Care level at baseline T_0_ was included to control for initial differences in care needs. Age and duration of residence were considered but showed weaker associations with outcomes than care level at T_0_ (see Table 1) and were omitted to reduce model complexity given the small sample sizes. While functionality was assessed by staff, analyses of affect, resilience, and sleep quality included report type as a control variable (SelfReport: 1 = *completed with the resident*, 0 = *completed by staff*) to account for differences between self- and proxy ratings.

### Statistical analyses

Data preparation and preliminary analyses (e.g. factor analyses, correlations) were conducted with IBM SPSS Statistics 29. All hypothesis tests (H_1_-H_5_) were performed in R 4.5.1 using the package lme4 [84]. For H_1_-H_4_, we estimated multilevel linear models with time points at Level 1 (within-person) and residents at Level 2 (between-person), including random intercepts for care facilities and residents. Model 1 included time and covariates as fixed effects; Model 2 added the time x group interaction. Models were compared using likelihood ratio tests. As including care facilities as Level-3 unit did not improve model fit or affect fixed-effect estimates, this level was not included in subsequent analyses.

For H_5_, analyses were restricted to the IG and required participation at T_0_. The number of received clown visits was decomposed into between- and within-person components using group-mean centring. We estimated Model 1 (covariates and time), Model 2 (adding both visit-frequency components), and Model 3 (adding the time x within-person visit frequency interaction). Model fit was again evaluated using likelihood ratio tests.

An a priori power analysis was conducted with G*Power [85] using a multiple regression model (*R² increase)* to approximate the planned multilevel analyses. Following preregistration, the analysis was updated to reflect the final model specification. Assuming a medium effect size (*f²* = 0.15 [18]), α = 0.05, and (1-β) = 0.80, approximately *N* = 290 participants are required to detect a medium-sized interaction (e.g. time x group) while including up to seven additional predictors. This estimate is conservative, as repeated measurements per participant are expected to increase power.

## Results

Table 1 displays the reliabilities and correlations of all variables at T_1_ (a correlation matrix including all time points is available in the supplementary material). Notably, the correlations within the individual IGs (vs. all other residents) vary in strength and direction, indicating heterogeneous associations with the outcome variables.

**Table 1:** Spearman correlation matrix of all variables in T_1_

Variable		*M*_IG_	*SD*_IG_	*M*_CG_	*SD*_CG_	1	2	3	4	5	6	7	8	9	10	11
1.	Care facility 1 (IG) ^a^	0.29	0.46	-	-	( - )	-	-	-	-	-	-	-	-	-	-
2.	Care facility 2 (IG) ^a^	0.25	0.44	-	-	-.37^**^	( - )	-	-	-	-	-	-	-	-	-
3.	Care facility 3 (IG) ^a^	0.46	0.50	-	-	-.58^**^	-.53^**^	( - )	-	-	-	-	-	-	-	-
4.	Care facility 4 (CG) ^a^	-	-	1.00	0.00	-	-	-	( - )	-	-	-	-	-	-	-
5.	Age T_1_	84.44	8.07	87.04	7.69	.18^*^	.11	-.25^**^	-	( - )	.13	.20	.22	.49^*^	.21	-
6.	Gender T_1_^b^	0.77	0.42	0.78	0.42	.07	.13	-.17^*^	-	.22^**^	( - )	.06	.24	-.16	-.01	-
7.	Duration of residence T_1_	2.93	2.67	3.77	2.23	.18^*^	-.10	-.08	-	-.09	.06	( - )	.35^*^	.33	.25	-
8.	Dementia stage T_1_^c^	1.70	1.34	1.40	1.48	-.12	-.36^**^	.43^**^	-	-.06	-.09	.06	( - )	.43^*^	.36^**^	-
9.	Care level at baseline T_0_^d^	3.43	0.96	3.00	0.71	-.06	-.18^*^	.21^*^	-	-.18^*^	.07	.21^*^	.45^**^	( - )	1.00^**^	-
10.	Care level T_1_^d^	3.59	0.88	3.34	0.75	-.15	-.18^*^	.30^**^	-	-.14	.07	.24^**^	.51^**^	.83^**^	( - )	-
11.	Frequency of clown visits year 1	9.37	8.64	0.00	0.00	.20^**^	-.40^**^	.18^*^	-	-.04	.12	.56^**^	.18^*^	.06	.20^**^	( - )
12.	Self-report T_1_^e^	0.26	0.44	0.65	0.48	.44^**^	-.21^*^	-.19^*^	-	-.01	-.01	.10	-.28^**^	-.41^**^	-.38^**^	.12
13.	Positive affect T_1_^f^	3.10	0.74	3.16	0.73	.45^**^	-.20^*^	-.22^**^	-	.21^**^	.09	-.05	-.24^**^	-.29^**^	-.26^**^	.15
14.	Negative affect T_1_^f^	2.37	0.66	2.44	0.70	-.34^**^	.38^**^	-.05	-	-.07	.07	-.06	.04	.22^*^	.14	-.21^*^
15.	Resilience T_1_^g^	3.55	1.43	4.40	1.22	.11	.35^**^	-.41^**^	-	.28^**^	.19^*^	-.15	-.45^**^	-.40^**^	-.40^**^	-.20^*^
16.	Sleep disturbance T_1_^h^	1.90	0.77	2.45	0.87	-.06	.40^**^	-.31^**^	-	.07	.13	-.19^*^	-.33^**^	-.26^**^	-.21^**^	-.35^**^
17.	Daytime sleepiness T_1_^h^	1.55	0.96	2.05	1.11	.12	-.03	-.08	-	-.02	.12	.04	.17^*^	.12	.13	-.07
18.	Overall sleep quality T_1_^i^	3.04	0.68	2.76	0.58	-.01	-.31^**^	.29^**^	-	-.15	-.15	.19^*^	.29^**^	.20^*^	.21^**^	.30^**^
19.	Selfcare difficulties T_1_^j^	2.69	0.97	2.44	1.08	-.16^*^	-.03	.17^*^	-	-.10	.11	.14	.48^**^	.65^**^	.65^**^	.02
20.	Disorientation T_1_^j^	2.53	1.01	2.36	1.06	-.07	-.34^**^	.36^**^	-	-.14	-.03	-.05	.81^**^	.48^**^	.51^**^	.17^*^
21.	Depression T_1_^j^	1.85	0.66	1.90	0.83	-.18^*^	.28^**^	-.08	-	-.10	.06	-.10	.04	.10	.10	-.14
22.	Irritability T_1_^j^	1.62	0.62	1.68	0.68	-.11	-.05	.14	-	-.14	-.11	.04	.39^**^	.39^**^	.34^**^	.06
23.	Withdrawal T_1_^j^	2.62	0.83	2.75	0.97	-.08	-.16^*^	.22^**^	-	-.10	-.09	.15	.57^**^	.52^**^	.52^**^	.08

Variable		12	13	14	15	16	17	18	19	20	21	22	23			
1.	Care facility 1 (IG) ^a^	-	-	-	-	-	-	-	-	-	-	-	-			
2.	Care facility 2 (IG) ^a^	-	-	-	-	-	-	-	-	-	-	-	-			
3.	Care facility 3 (IG) ^a^	-	-	-	-	-	-	-	-	-	-	-	-			
4.	Care facility 4 (CG) ^a^	-	-	-	-	-	-	-	-	-	-	-	-			
5.	Age T_1_	-.24	-.18	.05	-.15	.07	.30	-.07	.30^*^	.15	.02	-.09	.23			
6.	Gender T_1_^b^	-.25	.19	-.03	-.11	.01	-.06	-.11	-.03	.21	.05	.01	.00			
7.	Duration of residence T_1_	-.26	-.19	.34^*^	-.26	-.18	.20	.02	.17	.23	.08	.29^*^	.30^*^			
8.	Dementia stage T_1_^c^	-.67^**^	-.10	.22	-.57^**^	-.15	.20	.01	.59^**^	.77^**^	-.12	.29^*^	.59^**^			
9.	Care level at baseline T_0_^d^	-.56^*^	.02	.20	-.08	-.18	.44^*^	.04	.64^**^	.49^*^	.12	.07	.62^**^			
10.	Care level T_1_^d^	-.47^**^	-.01	.14	-.04	-.29	.38^*^	.21	.59^**^	.49^**^	-.19	.02	.53^**^			
11.	Frequency of clown visits year 1	-	-	-	-	-	-	-	-	-	-	-	-			
12.	Self-report T_1_^e^	( - )	.02	-.13	.51^**^	.11	-.44^*^	.07	-.48^**^	-.72^**^	.03	-.16	-.52^**^			
13.	Positive affect T_1_^f^	.39^**^	(.90/.83)	-.57^**^	.48^**^	-.20	-.17	.26	-.14	-.11	-.21	-.50^**^	-.36^*^			
14.	Negative affect T_1_^f^	-.37^**^	-.60^**^	(.81/.76)	-.63^**^	.37^*^	.33^*^	-.43^**^	.26	.41^**^	.55^**^	.24	.42^**^			
15.	Resilience T_1_^g^	.28^**^	.40^**^	-.31^**^	(.95/.89)	-.08	-.14	.31	-.40^*^	-.64^**^	-.45^**^	-.46^**^	-.61^**^			
16.	Sleep disturbance T_1_^h^	-.03	-.04	.22^**^	.22^**^	(.82/.74)	.15	-.73^**^	-.17	-.11	.29	-.10	-.18			
17.	Daytime sleepiness T_1_^h^	.04	-.26^**^	.20^*^	-.18^*^	.22^**^	( - )	-.18	.30	.41^*^	.41^*^	.09	.44^**^			
18.	Overall sleep quality T_1_^i^	-.02	.06	-.20^*^	-.21^**^	-.81^**^	-.30^**^	( - )	.12	-.04	-.40^*^	-.04	.01			
19.	Selfcare difficulties T_1_^j^	-.36^**^	-.33^**^	.20^*^	-.35^**^	-.12	.23^**^	.14	(.92/.93)	.60^**^	.06	.10	.78^**^			
20.	Disorientation T_1_^j^	-.29^**^	-.20^*^	.10	-.48^**^	-.30^**^	.18^*^	.29^**^	.50^**^	(.93/.94)	.12	.32^*^	.75^**^			
21.	Depression T_1_^j^	-.08	-.37^**^	.56^**^	-.17^*^	.30^**^	.26^**^	-.26^**^	.11	.11	(.79/.87)	.26	.16			
22.	Irritability T_1_^j^	-.22^**^	-.35^**^	.39^**^	-.40^**^	-.04	.14	.09	.25^**^	.44^**^	.39^**^	(.82/.83)	.27			
23.	Withdrawal T_1_^j^	-.38^**^	-.48^**^	.30^**^	-.49^**^	-.20^*^	.27^**^	.13	.57^**^	.66^**^	.06	.38^**^	(.88/.90)			

IG below diagonal/CG above diagonal. _*nT*__*1_IG*_ = 153-163; *n*_*T1_CG*_ = 39-50. Reliabilities (Cronbach's α) are shown diagonally in brackets.

* *p*<0.05 ** *p*<0.01

^a^ Care facilities are dummy-coded: 1 = member of the facility, 0 = all other residents, ^b^ 0 = men, 1 = women, ^c^ 1 = no dementia, 1 = early stage, 2 = moderate stage, 3 = late stage, 4 = end-stage dementia, ^d^ 1 = minor to 5 = severe impairments, ^e^ 1 = survey completed together with resident, 0 = survey completed by staff, ^f^ 1 = very rarely or never to 5 = very often or always, ^g^ 1 = not at all to 7 = fully, ^h^ 1 = not during the past month to 4 = three or more times a week, ^i^ 1 = very bad to 4 = very good, ^j^ 1 = behaviour is not present or is normal to 4 = behaviour is severe or occurs almost constantly.

Paired *t*-tests for the WCG (T_− 1_ to T_0_, see Table 6 in the Appendix revealed a significant increase in residents’ disorientation from T_− 1_ (*M* = 2.51, *SD* = 0.91) to T_0_ (*M* = 2.70, *SD* = 0.96, *t*(42 = -2.29, *d* = − 0.35, *p* = .03)) before the start of clown visits, whereas no significant changes were observed in the other variables. The results of the multilevel models analysing H_1_-H_4_ are displayed in Table 2.

**Table 2:** Multilevel model results testing H_1_ affect, H_2_ resilience, H_3_ sleep quality and H_4_ functionality

	Affect				Resilience		Sleep quality					
	Positive affect		Negative affect				Sleep disturbance		Daytime sleepiness		Sleep quality overall	
	Model 1	Model 2	Model 1	Model 2	Model 1	Model 2	Model 1	Model 2	Model 1	Model 2	Model 1	Model 2
(Intercept)	3.48^***^	3.61^***^	1.78^***^	1.79^***^	5.67^***^	5.88^***^	2.18^***^	1.98^***^	0.69^**^	0.35	2.75^***^	3.03^***^
Gender [female]	0.19^*^	0.19^*^	0.01	0.01	0.18	0.18	0.16	0.17	0.19	0.20	-0.12	-0.13
Care level T_0_	-0.10^*^	-0.09^*^	0.08	0.09^*^	-0.25^***^	-0.24^***^	-0.17^***^	-0.17^***^	0.15^**^	0.14^**^	0.10^*^	0.10^*^
SelfReport	-0.17^*^	-0.18^**^	0.22^**^	0.21^**^	-0.77^***^	-0.81^***^	0.12	0.14	0.22^*^	0.24^*^	-0.05	-0.07
Group [IG]	0.08	-0.07	0.05	0.01	0.14	-0.11	0.14	0.31	-0.08	0.30	-0.02	-0.34
Time Point [T_1_]	0.05	-0.03	-0.07	0.07	-0.05	-0.03	-0.01	0.59^**^	-0.01	0.68^**^	0.08	-0.44^**^
Time Point [T_2_]	0.05	-0.26	-0.26^***^	-0.48^**^	0.09	-0.58^*^	-0.03	-0.19	-0.31^**^	-0.12	0.01	-0.20
Group [IG] x Time Point [T_1_]		0.09		-0.17		-0.02		-0.72^***^		-0.84^***^		0.64^***^
Group [IG] x Time Point [T_2_]		0.39^*^		0.27		0.81^**^		0.22		-0.22		0.25
**Random Effects**												
σ^2^	0.26	0.26	0.28	0.27	0.69	0.66	0.48	0.45	0.64	0.63	0.33	0.32
τ_00_ Residents	0.15	0.14	0.14	0.14	0.30	0.31	0.15	0.16	0.17	0.16	0.09	0.09
τ_00_ Care facilities	0.12	0.11	0.05	0.06	0.18	0.19	0.06	0.06	0.00	0.00	0.04	0.04
Observations	462	462	460	460	452	452	466	466	464	464	462	462
Deviance (*-2LL*)	863.40	857.34	876.38	868.32	1255.00	1243.15	1093.14	1067.62	1201.49	1188.42	899.30	885.69
Change in deviance (*Δ-2LL*)		6.06*		8.06*		11.85**		25.52***		13.07**		13.61**
AIC	904.01	906.02	918.21	917.93	1290.04	1284.05	1132.62	1114.26	1243.73	1237.16	941.45	935.71

Functionality												
	Selfcare difficulties		Disorientation		Depression		Irritability		Withdrawal			
	Model 1	Model 2	Model 1	Model 2	Model 1	Model 2	Model 1	Model 2	Model 1	Model 2		
(Intercept)	-0.00	0.01	0.81	0.80	1.72^***^	1.57^***^	1.26^***^	1.28^***^	1.41^***^	1.37^***^		
Gender [female]	0.13	0.13	-0.05	-0.04	0.06	0.07	-0.17^*^	-0.17^*^	-0.21^*^	-0.20^*^		
Care level T_0_	0.68^***^	0.68^***^	0.45^***^	0.45^***^	0.01	0.00	0.15^***^	0.15^***^	0.40^***^	0.40^***^		
SelfReport												
Group [IG]	0.06	0.06	0.01	0.04	0.17	0.36	0.04	0.01	-0.13	-0.08		
Time Point [T_1_]	0.21^***^	0.08	0.10^*^	0.10	-0.06	0.29^*^	-0.01	0.06	0.14^*^	0.22		
Time Point [T_2_]	0.36^***^	0.49^***^	0.09	0.14	-0.32^***^	-0.27^*^	-0.14^**^	-0.27^*^	0.10	0.12		
Group [IG] x Time Point [T_1_]		0.16		0.01		-0.43^**^		-0.09		-0.10		
Group [IG] x Time Point [T_2_]		-0.17		-0.06		-0.04		0.17		-0.03		
**Random Effects**												
σ^2^	0.22	0.22	0.17	0.17	0.27	0.26	0.18	0.18	0.24	0.24		
τ_00_ Residents	0.38	0.38	0.58	0.58	0.20	0.21	0.20	0.20	0.33	0.33		
τ_00_ Care facilities	0.01	0.01	0.12	0.13	0.04	0.04	0.00	0.00	0.01	0.01		
Observations	535	535	535	535	535	535	535	535	535	535		
Deviance (*-2LL*)	1071.48	1063.15	1053.64	1053.11	1044.88	1031.52	877.03	871.13	1073.16	1072.51		
Change in deviance (*Δ-2LL*)		8.33^**^		0.53		13.36^**^		5.90		0.65		
AIC	1110.26	1110.83	1088.22	1096.98	1081.99	1077.24	919.70	923.09	1112.17	1120.12		

*N*_*Care_facilities*_= 4; *N*_*residents*_ = 211-22_0_. Group [IG] = intervention group (reference = CG). Time Point [T_0_] = baseline (reference category). Gender [female] = 1 (0 = male). Care level T_0_ = initial care level at baseline. SelfReport = resident’s self-assessment (0 = proxy report). Random effects were specified for residents and care facilities.

* *p*<0.05 ** *p*<0.01 *** *p*<0.001

### Affect

Model 1 showed no main effect over time for positive affect, but a decrease in negative affect from T_0_ to T_2_ (β = -0.26, *p* < .001). In Model 2, only positive affect showed a significant group x time interaction at T_2_ on the parameter level (β = 0.39, *p* < .05; *Δ-2LL* = 6.06*, *p* < .05), with a small effect size *(f²* = 0.01). H_1_ was therefore only partially supported, as the IG demonstrated a significantly more favourable trajectory in positive affect at T_2_ (H_1a_ii_) compared to the CG, with no corresponding effects for negative affect or other time points.

### Resilience

No significant main effects over time were found in Model 1. Model 2 revealed a significant group x time interaction at T_2_ (β = 0.81, *p* < .01; *Δ-2LL* = 11.85**, *p* < .01) with a small effect size *(f²* = 0.02). Consequently, H_2_ is supported only for ii) T_2_, indicating a smaller long-term decline in resilience in the IG compared to the CG after two years.

### Sleep quality

Model 1 revealed a significant main effect of reduced daytime sleepiness at T_2_ (β = -0.31, *p* < .01). In Model 2, significant group x time interactions were found for sleep disturbance at T_1_ (β = -0.72, *p* < .001; *Δ-2LL* = 25.52***, *p* < .001) with a small effect size (*f²* = 0.04), daytime sleepiness at T_1_ (β = -0.84, *p* < .001; *Δ-2LL* = 13.07**, *p* < .01) with a very small effect size (*f²* = 0.004), and overall sleep quality at T_1_ (β = 0.64, *p* < .001; *Δ-2LL* = 13.61**, *p* < .001) with a small effect size (*f²* = 0.02). Thus, H_3a−c_ are supported only for i) T_1_, suggesting that, after one year, sleep disturbances and daytime sleepiness increased less, and overall sleep quality declined less, in the IG compared to the CG.

### Functionality

Model 1 demonstrated significant main effects over time in increased self-care difficulties in both groups (IG & CG) at T_1_ (β = 0.21, *p* < .001) and T_2_ (β = 0.36, *p* < .001), and a small increase in disorientation at T_1_ (β = 0.10, *p* < .05). Depressed mood decreased at T_2_ (β = -0.32, *p* < .001), irritability also decreased at T_2_ (β = -0.14, *p* < .01), and withdrawal increased at T_1_ (β = 0.14, *p* < .01). In Model 2, only depression showed a significant group x time interaction at T_1_ (β = -0.43, *p* < .01; *Δ-2LL* = 13.36**, *p* < .01), with a small effect size (*f²* = 0.01). H_4_ was therefore only partially supported, as a significantly more favourable trajectory in depressive mood at T_1_ (H_4c_i_) was observed in the IG compared to the CG, but not for the other subscales or time points.

### Frequency of clown visits

Analyses for H_5_ were conducted within the IG only to examine whether the effects of clown visits on affect, resilience, sleep quality, and functionality were moderated by the frequency of visits. Model 1 replicated the time effects hypothesised in H_1_-H_4_, showing decreased negative affect, improved resilience and sleep quality, alongside higher self-care difficulties, withdrawal, and decreased depressive mood over time in the IG (see Table 3 for detailed results).

**Table 3:** Multilevel model results testing moderation by clown visit frequency H_5_

	Affect						Resilience			Sleep quality										
	Positive affect			Negative affect						Sleep disturbance				Daytime sleepiness				Sleep quality overall		
	Model 1	Model 2	Model 3	Model 1	Model 2	Model 3	Model 1	Model 2	Model 3	Model 1	Model 2	Model 3	Model 1		Model 2	Model 3	Model 1		Model 2	Model 3
(Intercept)	3.68^***^	3.59^***^	3.55^***^	1.59 ^***^	1.63 ^***^	1.63 ^***^	5.94^***^	5.59^***^	5.53^***^	2.09^***^	2.22^***^	2.24^***^	0.54		0.82^**^	0.89^**^	2.77^***^		2.60^***^	2.55^***^
Gender [female]	0.24^*^	0.19^*^	0.19	0.08	0.13	0.13	0.32^*^	0.31^*^	0.31^*^	0.23	0.26^*^	0.26^*^	0.20		0.21	0.21	-0.19		-0.22^*^	-0.22^*^
Care level T_0_	-0.13^**^	-0.13^**^	-0.13^**^	0.14 ^**^	0.13 ^**^	0.13 ^**^	-0.30^***^	-0.29^***^	-0.29^***^	-0.14^*^	-0.15^**^	-0.15^**^	0.17^*^		0.17^*^	0.17^*^	0.09		0.10^*^	0.10^*^
SelfReport	-0.22^**^	-0.22^**^	-0.22^**^	0.22 ^**^	0.24 ^**^	0.24 ^**^	-0.86^***^	-0.81^***^	-0.81^***^	0.16	0.15	0.15	0.26^*^		0.21	0.21	-0.05		-0.04	-0.03
Time point [T_1_]	0.03	-0.02	0.06	-0.06	0.05	0.10	-0.11	0.15	0.23	-0.16	-0.20	-0.16	-0.13		-0.30^*^	-0.39^*^	0.18^*^		0.26^*^	0.29^*^
Time point [T_2_]	0.14	0.05	0.08	-0.21 ^**^	-0.02	-0.06	0.25^*^	0.66^***^	0.72^***^	0.05	0.01	-0.04	-0.34^**^		-0.59^***^	-0.67^***^	0.04		0.14	0.22
Frequency clown visits between		0.02^*^	0.01		-0.02 ^**^	-0.03 ^**^		0.01	0.00		-0.01	-0.01			-0.01	0.00			0.01	0.01
Frequency clown visits within		0.00	-0.01		-0.01	-0.02		-0.03^**^	-0.04^*^		0.00	0.00			0.02^*^	0.04^*^			-0.01	-0.02
Time point [T_1_] x Frequency clown visits within			0.02			0.00			0.03			-0.01				-0.04				0.03
Time point [T_2_] x Frequency clown visits within			0.02			0.01			0.03			0.01				-0.03				0.01
**Random Effects**																				
σ^2^	0.24	0.24	0.24	0.28	0.28	0.28	0.72	0.67	0.68	0.42	0.42	0.43	0.61		0.60	0.60	0.33		0.33	0.34
τ_00_ Residents	0.13	0.12	0.12	0.12	0.11	0.12	0.22	0.25	0.24	0.15	0.15	0.15	0.27		0.28	0.27	0.09		0.08	0.08
τ_00_ Care Facilities	0.13	0.12	0.12	0.07	0.03	0.03	0.18	0.19	0.20	0.05	0.04	0.03	0.00		0.00	0.00	0.04		0.02	0.02
Observations	320	320	320	320	320	320	319	319	319	321	321	321	320		320	320	317		317	317
Deviance (*-2LL*)	562.60	555.85	553.78	597.22	585.90	585.05	872.92	863.81	862.53	718.13	715.68	715.12	840.62		834.48	832.54	615.37		610.26	608.24
Change in deviance (*Δ-2LL*)		6.75^*^	2.07		11.32^**^	0.85		9.11^**^	1.28		2.45	0.56			6.14^*^	1.94			5.11	2.02
AIC	599.52	613.50	628.28	634.32	644.06	659.84	904.75	914.21	927.70	753.58	771.01	786.22	875.83		888.70	901.82	652.59		668.13	682.47

	Functionality																			
	Selfcare difficulties			Disorientation			Depression			Irritability			Withdrawal							
	Model 1	Model 2	Model 3	Model 1	Model 2	Model 3	Model 1	Model 2	Model 3	Model 1	Model 2	Model 3	Model 1	Model 2	Model 3					
(Intercept)	0.08	0.17	0.14	0.85^*^	0.70^*^	0.70^*^	1.82^***^	1.88^***^	1.86^***^	1.10^***^	1.05^***^	1.04^***^	1.44^***^	1.56^***^	1.61^***^					
Gender [female]	0.01	0.01	0.00	-0.12	-0.17	-0.17	0.15	0.18	0.17	-0.20^*^	-0.20^*^	-0.20^*^	-0.34^**^	-0.33^**^	-0.33^**^					
Care level T_0_	0.70^***^	0.70^***^	0.70^***^	0.45^***^	0.46^***^	0.46^***^	0.02	0.02	0.02	0.21^***^	0.21^***^	0.21^***^	0.39^***^	0.39^***^	0.39^***^					
Time point [T_1_]	0.23^***^	0.10	0.19^*^	0.09	0.00	-0.02	-0.14^*^	-0.07	-0.05	-0.04	0.01	0.01	0.14^*^	0.03	-0.10					
Time point [T_2_]	0.33^***^	0.13	0.10	0.08	-0.07	-0.06	-0.29^***^	-0.18	-0.14	-0.09	-0.02	-0.01	0.09	-0.08	-0.07					
Frequency clown visits between		0.00	-0.01		0.03 ^*^	0.03^*^		-0.02	-0.02		0.00	0.00		0.00	0.01					
Frequency clown visits within		0.01^**^	0.00		0.01 ^*^	0.01		-0.01	-0.01		0.00	-0.01		0.01^*^	0.03^**^					
Time point [T_1_] x Frequency clown visits within			0.02			0.00			0.02			0.00			-0.03					
Time point [T_2_] x Frequency clown visits within			0.03			0.00			0.01			0.00			-0.04^*^					
**Random Effects**																				
σ^2^	0.21	0.20	0.20	0.15	0.15	0.15	0.25	0.25	0.25	0.17	0.17	0.17	0.23	0.22	0.22					
τ_00_ Residents	0.33	0.33	0.33	0.55	0.54	0.54	0.21	0.21	0.21	0.20	0.20	0.20	0.29	0.29	0.30					
τ_00_ Care Facilities	0.02	0.02	0.02	0.12	0.06	0.06	0.07	0.05	0.06	0.00	0.00	0.00	0.00	0.00	0.01					
Observations	356	356	356	356	356	356	356	356	356	356	356	356	356	356	356					
Deviance (*-2LL*)	675.90	669.18	665.42	663.22	652.22	652.08	680.49	675.76	674.94	567.07	566.17	566.05	680.26	675.74	670.18					
Change in deviance (*Δ-2LL*)		6.72^*^	3.76		11.00^**^	0.14		4.73	0.82		0.90	0.12		4.52	5.56					
AIC	708.73	722.21	735.79	694.53	704.33	721.88	712.40	728.27	744.25	602.77	622.89	640.35	714.03	729.03	741.02					

*N*_*Care_facilities*_ = 4; *N*_*Residents*_ = 137-139. Gender [female] = 1 (0 = male). Care level T_0_= initial care level at baseline. SelfReport = resident’s self-assessment (_0_ = proxy report). Time Point [T_0_] = baseline (reference category). Random effects were specified for residents and care facilities

* *p*<0.05 ** *p*<0.01 *** *p*<0.001

In Model 3, the time x frequency visits within-person interaction for withdrawal at T_2_ reached parameter-level significance (β = -0.04, *p* < .05), indicating that within individuals, higher visit frequency was associated with increased withdrawal at T_2_; however, this interaction did not improve overall model fit (*Δ-2LL* = 5.56, *p* = .06) and should therefore be considered as a trend. No other moderation effects emerged; thus, H_5_ is not supported.

Beyond the hypothesised moderation effects, Model 2 revealed several main associations between visit frequency and residents’ emotional, sleep-related and functional outcomes. At the within-person level, a higher frequency of clown visits was associated with lower resilience, but also with greater daytime sleepiness, self-care difficulties, disorientation, and withdrawal. At the between-person level, residents who experienced a higher average frequency of visits tended to show higher positive affect, lower negative affect, and greater disorientation. These findings should be interpreted exploratively.

## Discussion

This study provides a controlled longitudinal evaluation of clown visits in long-term care over two years. Time-specific benefits emerged for emotional well-being, sleep-related outcomes, and resilience, whereas broader functional trajectories remained largely unchanged. Improvements were most evident after one year for depressive mood and sleep quality and after two years for positive affect and resilience. Visit frequency did not moderate these effects, pointing to context- and need-related delivery patterns rather than a dose-response relationship.

### Affect

Negative affect declined in both groups over the two-year period, whereas positive affect showed no overall change but a more favourable trajectory in the IG than in the CG. This pattern is consistent with Lawton’s dual-channel hypothesis, which posits that negative affect is less responsive to external stimulation, whereas positive affect is more susceptible to externally engaging interventions [86]. Correspondingly, similar effects have been observed for individualised psychosocial interventions delivered by care staff [49], and short-term experimental studies indicate that clown interventions primarily elicit increases in positive affect without reducing negative mood [87]. Taken together, these findings suggest selective effects on positive affective components.

### Resilience

Although no significant main effects emerged across the two-year period, a significant group-by-time interaction indicated that resilience declined less in the IG than in the CG at T_2_. This finding is consistent with the Broaden-and-Build theory [23, 27], as the trend toward strengthened positive affect in the IG corresponds with the observed resilience pattern after two years. Despite the small effect, the findings provide one of the first longitudinal indications that resilience among residents can be sustained through medical clowning in long-term care.

### Sleep quality

Residents in the IG showed lower sleep disturbance, reduced daytime sleepiness, and better overall sleep quality after one year compared to the CG, consistent with previous evidence that humour-based interventions can improve sleep-related outcomes [68, 70]. Although reduced daytime sleepiness was also observed at T_2_, a similar reduction in the CG and the absence of an interaction effect preclude interpretation as a sustained intervention effect.

Several mechanisms may contribute to these sleep improvements, including physiological stress reduction through laughter [28], cognitive reframing of emotionally challenging experiences [35] and increased social connectedness in line with stress-buffering models [36, 37], although the specific pathways remain to be clarified. Given the high prevalence of sleep problems in long-term care and their association with depression, functional decline [88], and cognitive impairment [89], the findings suggest that clown visits may represent a valuable, albeit temporary, addition to care routines. However, as sleep in long-term care is strongly influenced by structural, health-related, and routine factors [88, 90], the persistence of such effects over longer periods may be limited.

### Functionality

Consistent with an age-related functional decline [7, 73], self-care difficulties increased in both groups over the two-year period, while disorientation showed a small increase after one year. Notably, this increase in disorientation had already emerged in the WCG prior to the intervention, indicating a general time-related trend rather than an intervention effect. In contrast, depressive mood showed a more pronounced decline in the IG after one year, representing the only significant group-by-time interaction. Depressive mood further decreased within the IG after two years, although without a lasting interaction effect. After two years, irritability had also decreased across both groups, indicating a general improvement unrelated to the intervention, whereas withdrawal increased after one year, consistent with age- or context-related behavioural change.

Overall, clown visits did not produce consistent effects across functional facets, except for depressive mood, suggesting a stronger influence on emotional than on physical, cognitive, or behavioural aspects shaped by age-related and care-context factors in long-term care settings [72, 73]. Importantly, the observed improvement in depressive mood is relevant in itself, as emotions and depressive symptoms are closely linked to activities of daily living in older adults [91, 92]. Thus, even in the absence of direct effects on other functional domains, changes in depressive mood highlight a potentially meaningful pathway through which clown visits may contribute to residents’ overall functional well-being.

### Frequency of clown visits

Contrary to expectations, individual-based visit frequency did not moderate changes in affect, resilience, sleep quality, or functionality over time, consistent with evidence that positive affect in nursing home residents relates more to activity variety than frequency [47]. Although the Broaden-and-Build theory posits that repeated positive experiences may foster upward spirals of well-being [26, 27], the present results suggest that frequency alone may not adequately capture the impact of clown visits.

Exploratory within-person analyses showed that higher visit frequency was associated with greater self-care difficulties, disorientation, withdrawal, lower resilience, and higher daytime sleepiness. These patterns may reflect adaptive delivery processes in long-term care, where clown interactions are embedded in everyday routines and occur situationally in shared or group-based settings rather than as fixed one-to-one sessions (e.g [17, 18]), allowing for flexible, context-sensitive engagement with residents showing greater psychosocial or functional needs.

Complementarily, at the between-person level, a higher average frequency of clown visits was associated with higher positive and lower negative affect, as well as greater disorientation. This pattern suggests that residents receiving more frequent clown visits tend to show more favourable affective states, while also differing in cognitive functioning. Similar associations between clown visits and affective outcomes have been reported in previous studies, particularly with respect to short-term affective responses (e.g [16, 38, 87]).

Taken together, the findings indicate that the effects of clown visits in long-term care are less dependent on individual-based visit frequency per se than on the contextual, relational, and emotional quality of the interactions, underscoring the importance of how such interventions are delivered.

### Strengths and limitations

A major strength of this study is its longitudinal, controlled field design with three measurement occasions over two years, including both a CG and an additional WCG, allowing intervention effects to be distinguished from general time trends. However, comparing three intervention facilities with a single control facility constitutes a limitation. Additionally, the field setting involves substantial contextual heterogeneity, as outcomes in long-term care are shaped by organisational factors, staff changes and external stressors such as the COVID-19 pandemic (e.g [93, 94]). Consequently, the intervention facilities responded differently to clown visits, with partly opposing associations across sites, potentially attenuating average intervention effects.

Using an adaptive combination of self- and proxy-report measures increased inclusiveness, while also involving potential common method bias in self-reports [95] and perspective-related discrepancies in proxy ratings [96]. The handling of *does not apply* (5) responses in the MOSES scale followed established recommendations to preserve statistical power [83], although this decision may have influenced scale properties [97].

Finally, multilevel modelling and the separation of within- and between-person effects represent analytical strengths. However, the achieved sample size was considerably smaller than the a priori estimated sample size, resulting in limited power, particularly for moderation analyses. Consequently, statistically significant findings may be regarded as conservative estimates, whereas non-significant effects should be interpreted as inconclusive rather than definitive null effects. At the same time, although the main hypotheses were preregistered and theory-driven, the study included multiple outcomes and exploratory follow-up analyses. Therefore, individual findings should be interpreted cautiously with regard to potential Type I error inflation. Generalisability beyond German long-term care settings should therefore be interpreted cautiously.

### Theoretical and practical implications

Accordingly, replication in larger, adequately powered samples is essential to clarify the robustness and scope of intervention effects. Moreover, heterogeneous patterns across intervention facilities suggest that the effects of clown visits in long-term care are strongly context-dependent, underscoring the need to examine organisational and situational moderators (e.g [98]). Further, the observed need-oriented and differential associations point to resident characteristics, such as cognitive and functional status or baseline affect, as potential moderators of effectiveness (e.g [99, 100]). Notably, the absence of frequency-related longitudinal effects highlights a key research gap concerning which qualitative features of clown visits, including interaction quality, duration, and mode of delivery, are most relevant for beneficial outcomes (e.g [47]). Beyond resident-level outcomes, the relational and emotional nature of clown interactions suggests that indirect effects on staff and staff-resident relationships may be a promising area for future research.

From a practical perspective, clown visits may serve as a low-threshold, preventive resource to promote residents’ emotional well-being, resilience and social participation in long-term care, consistent with the WHO’s UN Decade of Healthy Ageing 2021–2030 [1]. The findings suggest that individual-based visit frequency alone may be insufficient to capture their practical relevance, pointing instead to the potential importance of interaction quality and everyday integration. Moreover, the observed need-oriented patterns indicate that clown visits may naturally reach residents with greater psychosocial or functional vulnerability, highlighting their potential as a flexible intervention embedded in routine care without formal targeting. Accordingly, more favourable emotional states and resilience among residents may support core nursing care goals and, as suggested by previous research (e.g [6, 101]), be indirectly relevant for everyday nursing care demands and the work environment of care staff.

## Conclusion

This longitudinal evaluation suggests time-specific effects of clown visits in long-term care, with more favourable developments in depressive mood and sleep quality after one year and a reduced decline in positive affect and resilience after two years compared to a CG, independent of individual-based visit frequency. Exploratory analyses revealed associations between visit frequency and resident characteristics: residents with greater psychosocial or functional needs tended to receive more frequent visits, and higher visit frequency co-occurred with better mood alongside greater disorientation, suggesting adaptive delivery in response to resident needs. Overall, clown visits may represent a multimodal psychosocial intervention without adverse effects and with preventive potential to sustain residents’ emotional well-being and resilience, while potentially supporting everyday care work.

## Supplementary Information

Supplementary Material 1.

## Data Availability

The data that support the findings of this study are not publicly available due to ethical and data protection restrictions but are available from the corresponding author upon reasonable request.

## Appendix

**Table 4:** Sociodemographic characteristics

	Intervention group						Control group					
		*N*		*M (SD)*	Min	Max		*N*		*M (SD)*	Min	Max
	Valid	Missing	Percent				Valid	Missing	Percent			
Age	163	0		84.44 (8.07)	65	106	50	0		87.04 (7.69)	70	100
Gender	163	0		0.77 (0.42)			50	0		0.78 (0.42)		
Female	125		76.7				39		78.0			
Male	38		23.3				11		22.0			
Other	0		0.0				0		0			
Care level ^a^	163	0		3.59 (0.88)	1	5	50	0		3.34 (0.75)	2	5
Level 1	1		0.6				0		0.0			
Level 2	18		11.0				6		12.0			
Level 3	50		30.7				23		46.0			
Level 4	72		44.2				19		38.0			
Level 5	22		13.5				2		4.0			
Dementia level	163	0		1.70 (1.34)	0	4	50	0		1.40 (1.48)	0	4
No dementia	51		31.3				22		44.0			
Early stage	12		7.4				6		12.0			
Moderate stage	48		29.4				8		16.0			
Late stage	39		23.9				8		16.0			
End-stage dementia	13		8.0				6		12.0			
Duration of residence	161	2		2.93 (2.67)	0.02	12.5	50	0		3.77 (2.23)	0.5	8.5

Intervention group (IG): *N*_*T1*_ = 163; control group (CG): *N*_*T1*_ = 50. ^a^ Official German Pflegegrad (from Level 1 = minor to Level 5 = severe impairments)

**Table 5:** Handling of the response option (5) “*Does not apply*” in functionality assessment

			T_WG_			T_0_			T_1_			T_2_		
Subscale	Variable	5 becomes…	*n*	^N^	%	*n*	*N*	%	*n*	*N*	%	*n*	*N*	%
Disorientation	II-11: Finding way around inside	1	8	43	18.2	26	160	16.3	42	210	20.0	39	164	23.8
	II-13: Awareness of place	4	7	43	15.9	27	160	16.9	50	209	23.9	48	163	29.4
	II-14: Awareness of time	4	6	43	13.6	25	159	15.7	49	209	23.4	51	163	31.3
	II-15: Memory of recent events	4	6	43	13.6	23	161	14.3	48	209	23.0	45	163	27.6
	II-10: Talking	4	4	43	9.1	14	162	8.6	22	209	10.5	20	162	12.3
Depression	III-17: Looking sad and depressed	1	1	43	2.3	6	160	3.8	9	210	4.3	6	163	3.7
	III-19: Sounding sad and depressed	1	2	43	4.5	11	160	6.9	17	210	8.1	12	163	7.4
	III-18: Reporting sadness and depression	1	3	43	6.8	17	160	10.6	33	210	15.7	22	164	13.5
	III-21: Reporting worry and anxiety	1	3	43	6.8	17	161	10.6	33	210	15.7	22	163	13.5
	III-23: Pessimism about the future	1	5	43	11.4	20	160	12.5	41	210	19.5	29	164	17.7
Irritability	IV-29: Verbal abuse of staff	1	2	42	4.5	7	161	4.3	12	210	5.7	10	160	6.3
	IV-30: Verbal abuse of other residents	1	4	43	9.1	10	162	6.2	15	219	7.2	12	162	7.4
	IV-26: Following staff requests and instructions	4	2	43	4.5	13	161	8.1	27	210	12.9	21	162	13.0
Withdrawal	IV-40: Helping other residents	4	8	43	12.2	27	160	16.9	48	208	23.1	47	164	28.7
	IV-36: Friendships with other residents	4	2	43	4.5	20	159	12.6	35	210	16.7	20	164	12.2

The response option (5) *does not apply *was recoded following an established procedure [83], with values interpreted either as the highest impairment (4) or as not at all (1), depending on the functional relevance of the item. *n* = number of residents rated‘5 = does not apply ’ for the respective item; *N* = total number of residents assessed for the respective item

**Table 6:** Results paired *t*-tests comparing the waiting control group at T-_1_ and T_0_

Variable	*n*	*M*null (*SD*_T-1_)	*M*_T0_(*SD*_T0_)	*T*	*df*	*p*	*Cohen's d*
Positive affect	43	3.19 (0.78)	3.21 (0.79)	-0.22	42	.83	-.03
Negative affect	43	2.38 (0.67)	2.27 (0.68)	0.93	42	.36	.14
Resilience	43	3.75 (1.39)	3.89 (1.48)	-0.75	41	.46	-.12
Sleep disturbance	41	1.85 (0.68)	1.86 (0.81)	-0.10	40	.92	-.02
Daytime sleepiness	40	1.63 (0.98)	1.50 (0.93)	0.80	39	.43	.13
Sleep quality overall	40	3.18 (0.55)	3.13 (0.61)	0.40	39	.69	.06
Self-care difficulties	43	2.49 (1.02)	2.50 (0.97)	-0.06	42	.95	-.01
Disorientation	43	2.51 (0.91)	2.70 (0.96)	-2.29*	42	.03	-.35
Depression	43	2.06 (0.78)	1.95 (0.80)	0.89	42	.38	.14
Irritability	43	1.68 (0.68)	1.59 (0.61)	0.82	42	.42	.13
Withdrawal	43	2.45 (0.77)	2.50 (0.82)	-0.66	42	.51	-.10

T_-1_= three months prior to baseline T_0_(i.e. prior to the start of the clown visits) ** p<0.05*
